# Do errors in the GHQ-12 response options matter?

**DOI:** 10.1371/journal.pone.0314915

**Published:** 2024-12-05

**Authors:** Bethany Croak, Rupa Bhundia, Danielle Lamb, Neil Greenberg, Sharon A. M. Stevelink, Nora Trompeter, Simon Wessely, G. James Rubin

**Affiliations:** 1 Department of Psychological Medicine, King’s College London, London, United Kingdom; 2 Department of Applied Health Research, NIHR ARC North Thames, University College London, London, United Kingdom; UCL: University College London, UNITED KINGDOM OF GREAT BRITAIN AND NORTHERN IRELAND

## Abstract

**Background:**

The twelve item General Health Questionnaire (GHQ-12) is a widely used measure of psychological wellbeing. Because there are seven different sets of response options across the twelve items, there is scope for transcription errors to occur when researchers assemble their study materials. The impact of such errors might be more important if they occur in the first set of response options than if they occur later in the questionnaire, once participants have become aware that options to the right of the GHQ-12 response sets always indicate worse wellbeing.

**Aims:**

To test the impact of introducing errors into the first and eighth set of response options for the GHQ-12 that render those response sets partially illogical.

**Methods:**

We used a double-blind randomised controlled trial, pre-registered with Open Science Framework (osf.io/syhwf). Participants were recruited by a market research company from their existing panel of respondents in Great Britain. Participants were randomly allocated to receive one of three versions of the GHQ-12: a correct version (n = 500), a version with a mistake in the first item (n = 502), or a mistake in the eighth item (n = 502). Mistakes replaced ‘better than usual’ (item one) or ‘more so than usual’ (item eight) with ‘not at all.’

**Results:**

We found no differences between the versions in terms of number of participants with possible poor psychological wellbeing (χ^2^ = 0.32, df = 2, p = 0.85) or in mean GHQ-12 scores for the three groups (F(2, 1501) = 0.26, p = 0.77).

**Conclusions:**

Small deviations from the standard GHQ-12 wording do not have a substantive impact on results.

## Introduction

The agreed consensus is that validated questionnaires should not be modified without checking the impact on results [1], ideally by randomly allocating participants to receive variations of the item or scale [2].

The 12-item General Health Questionnaire (GHQ-12) is a measure of psychological wellbeing that is widely used in occupational health research and was a popular measure of psychiatric morbidity during the COVID-19 pandemic and continues to be, especially in healthcare workers [3–5]. Each item presents a psychological symptom and asks respondents to tick one of four responses. Responses suggesting worse wellbeing are always presented to the right of the scale. Seven sets of response options are used across the 12 items, with subtle differences in wording between these sets. The use of different response sets increases the likelihood of human error occurring when researchers transcribe the original scale in print for use in their own digital questionnaires. We have previously made such an error [6]. If these errors do indeed impact the results, this could lead to an under or over-representation of the prevalence of probable mental disorders in the chosen population. Thereofre, an investigation into the consequences of transcription errors on the GHQ-12 was important to measure the impact of our own error but also to inform future researchers who might face the same challenge.

One could hypothesise errors may have greater impact if they affect an item that appears early in the GHQ-12. As participants progress through later items, they may learn ticking a response to the right always indicates worse wellbeing, diminishing the importance of precise wording.

In this study, we tested whether introducing an error into the response options for items that appear early or late in the GHQ-12 leads to changes to the overall score or the proportion of participants meeting the criteria as a possible case of mental illness.

## Methods

A double-blind randomised controlled trial was conducted, pre-registered with Open Science Framework (osf.io/syhwf).

Ethical approval was given by King’s College London’s Research Ethics Committee (HR-23/24-39719). A market research company collected the data (between 30.01.2024 and 08.02.2024), using a pre-existing participant panel, representative of people in Great Britain in terms of age, gender and region. Participants earn points for surveys, exchangeable for a bank transfer (approximately 50p per survey). This panel had previously consented to complete omnibus surveys for the market research company. After the omnibus survey, an information sheet detailing this study was presented. Participants were informed they would be asked to complete a measure of psychological wellbeing for a university study, they were not told that the questionnaire might contain and error or the true purpose of the study. Participants were asked to provide consent for this study by tick a box confirming they agreed to continue and complete more questions for this study. If they agreed, the GHQ-12 versions were displayed to participants. At the end of the GHQ-12 questionnaire, they were provided with a debrief which explained the true purpose of the study; participants were given the opportunity to withdraw their data at that point.

Using simple randomisation, participants were alternately allocated by survey software to receive one of three versions of the GHQ-12 questionnaire. All participants received the survey link at the same time, and logged in at a time of their choice, thus producing an effective method of randomisation. No personnel were involved in assignment and participants were blinded. The researcher conducting the analysis was blind to group details until analysis completion.

Participants received one of three versions of the GHQ-12: the correct version, one with an error in the eighth item and one with an error in the first item. Errors replaced ‘better than usual’ (item one) or ‘more so than usual’ (item eight) with ‘not at all’ (see S1 Table). An error in different stages of the questionnaire (item one and eight) was included to test whether the position of the error had any effect, as we hypothesised that as participants progressed through the survey, they would have learnt that responses on the left indicated better wellbeing. Therefore, they would continue to answer on the basis of this logic, making the wrong wording in item eight void.

The GHQ-12 was scored using the 0-0-1-1 method, whereby the first two response options (indicating positive wellbeing) score 0, and the other two response options (indicating poorer wellbeing) score 1. A total score out of 12 (with a higher score indicating poorer wellbeing) was given with the standard cut-off score of four.

Using UK population norms [7], a sample size of 500 per group was deemed sufficient to detect a difference of one-point between two conditions at the 5% significance level with 99% power and, for GHQ-12 caseness, to detect a difference of six percentage points or more between the two conditions at the 5% significance level with 80% power.

Socio-demographic information collected included gender, age, ethnicity, educational attainment, region, socioeconomic status (defined by the occupational class of the chief household earner [8]) and Indices of Multiple Deprivation (IMD) quartile. IMD is a summary measure of relative deprivation informed by seven domains: income, employment, education, crime, housing, health and living environment. The first quintile indicating most deprived area and the fifth quintile indicating the least deprived [9].

Three ethnicity categories were analysed: White British/Welsh/Scottish/Northern Irish/British, Any other white background and Mixed/Asian/Black/Other. Further disaggregation was not possible due to low cell count.

Chi-square tests were used to test for significant differences in socio-demographic characteristics between groups. For gender, educational attainment, and socioeconomic status, ‘other’ or ‘prefer not to say’ were coded as missing due to low expected frequencies.

A one-way ANOVA was used to test for differences in total GHQ-12 score between the three groups. Chi-square tests were used to test for differences in the proportions meeting the cut-off in each group.

## Results

The socio-demographic characteristics of the sample (n = 1504) are summarised in Table 1. Chi-square tests of independence revealed no significant differences between the participant groups in gender ((χ^2^ (2), n = 1488) = 1.15, p = 0.56), ethnicity ((χ^2^ (4), n = 1504) = 2.70, p = 0.61), region ((χ^2^ (20), n = 1504) = 20.61, p = 0.42), educational attainment ((χ^2^ (12), n = 1444) = 6.12, p = 0.91), socioeconomic grade ((χ^2^ (10), n = 1501) = 7.19, p = 0.71) or IMD quartile ((X^2^ (6), n = 1504) = 6.71, p = 0.35).

**Table 1:** Socio-demographic characteristics of participants, according to whether they viewed the correct GHQ-12 or a version with an error in item one or eight.

Baseline characteristic	Correct GHQ-12	Error in item one	Error in item eight	Full sample
	n (%)	n (%)	n (%)	n (%)
***Gender***				
Man (including trans man)	212 (42%)	226 (45%)	211 (42%)	649 (43%)
Woman (including trans woman)	286 (57%)	270 (54%)	283 (56%)	839 (56%)
Other (including non-binary)	0 (0%)	2 (<1%)	3 (<1%)	5 (<1%)
Prefer not to say	2 (<1%)	4 (<1%)	5 (1%)	11 (<1%)
***Age***				
18 to 24	39 (8%)	46 (9%)	45 (9%)	130 (9%)
25 to 34	82 (16%)	90 (18%)	89 (18%)	261 (17%)
35 to 44	68 (14%)	60 (12%)	70 (14%)	198 (13%)
45 to 54	88 (18%)	73 (15%)	73 (15%)	234 (16%)
55 to 64	103 (21%)	101 (20%)	84 (17%)	288 (19%)
65 to 74	91 (18%)	95 (19%)	112 (22%)	298 (20%)
75+	29 (6%)	37 (7%)	29 (6%)	95 (6%)
***Ethnicity*** ^***a***^				
White English / Welsh / Scottish / Northern Irish / British	418 (84%)	422 (84%)	420 (84%)	1260 (84%)
Other White background	25 (5%)	29 (6%)	35 (7%)	89 (6%)
Mixed, Asian, Black or other	57 (11%)	51 (10%)	47 (9%)	155 (10%)
***Region***				
East of England	48 (10%)	52 (10%)	57 (11%)	157 (10%)
East Midlands	37 (7%)	35 (7%)	43 (9%)	115 (8%)
London	50 (10%)	59 (12%)	64 (13%)	173 (12%)
North East	14 (3%)	28 (6%)	20 (4%)	62 (4%)
North West	60 (12%)	52 (10%)	53 (11%)	165 (11%)
South East	66 (13%)	78 (16%)	59 (12%)	203 (14%)
South West	46 (9%)	37 (7%)	39 (8%)	122 (8%)
West Midlands	50 (10%)	53 (11%)	41 (8%)	144 (10%)
Yorkshire and The Humber	50 (10%)	29 (6%)	41 (8%)	120 (8%)
Scotland	49 (10%)	52 (10%)	52 (10%)	153 (10%)
Wales	30 (6%)	27 (5%)	33 (7%)	90 (6%)
***Educational Attainment***				
No qualifications	25 (5%)	24 (5%)	28 (6%)	77 (5%)
Up to 4 GCSEs or equivalent (NVQ level 1)	80 (16%)	73 (15%)	77 (15%)	230 (15%)
5 or more GCSEs or equivalent (NVQ level 2)	59 (12%)	65 (13%)	63 (13%)	187 (12%)
A levels or equivalent (Such as Scottish Highers or NVQ level 3)	122 (24%)	115 (23%)	115 (23%)	352 (23%)
Bachelors Degree or equivalent (such as HND or NVQ level 4)	137 (27%)	139 (28%)	132 (26%)	408 (27%)
Masters Degree or equivalent (NVQ level 5)	58 (12%)	50 (10%)	63 (13%)	171 (11%)
PhD	5 (1%)	10 (2%)	4 (<1%)	19 (1%)
Other qualification	12 (2%)	20 (4%)	13 (3%)	45 (3%)
Prefer not to say	2 (<1%)	6 (1%)	7 (1%)	15 (1%)
***Socioeconomic Grade***				
A–High managerial, administrative or professional	43 (9%)	36 (7%)	51 (10%)	130 (9%)
B–Intermediate managerial, administrative or professional	110 (22%)	121 (24%)	105 (21%)	336 (22%)
C1—Supervisory, clerical and junior managerial, administrative or professional	125 (25%)	124 (25%)	134 (27%)	383 (26%)
C2 –Skilled manual workers	85 (17%)	77 (15%)	66 (13%)	228 (15%)
D–Semi and unskilled manual workers	56 (11%)	62 (12%)	63 (13%)	181 (12%)
E–State pensioners, casual or lowest grade workers, unemployed with state benefits only	79 (16%)	82 (16%)	82 (16%)	243 (16%)
Prefer not to say	2 (<1%)	0 (0%)	1 (<1%)	3 (<1%)
***Index of Multiple Deprivation Quartile***				
Least deprived	109 (22%)	134 (27%)	109 (22%)	352 (23%)
2	109 (22%)	118 (24%)	125 (25%)	352 (23%)
3	141 (28%)	121 (24%)	133 (27%)	395 (26%)
Most deprived	141(28%)	129 (26%)	135 (27%)	405 (27%)
***TOTAL***	500 (33%)	502 (33%)	502 (33%)	1504 (100%)

^a^ Other white background includes: Irish, Gypsy or Irish Traveller, Any other white background. Mixed, Asian, Black or other includes: White and Black Caribbean, White and Black African, White and Asian, Any other Mixed/Multiple ethnic backgrounds, Indian, Pakistani, Bangladeshi, Chinese, Any other Asian background, Caribbean, African, Any other Black/African/Caribbean background, Arab and Other

The mean GHQ-12 score for the whole sample was 3.66. Group mean scores are described in Table 2. A one-way ANOVA revealed no significant difference in GHQ-12 scores between groups (F (2, 1501) = 0.26, p = 0.77).

**Table 2:** Mean GHQ-12 scores and proportion of cases by group, according to whether they viewed the correct GHQ-12 or a version with an error in item one or eight.

	Correct GHQ-12	Error in item one	Error in item eight	Full sample
Mean GHQ-12 score (SD)	3.76 (3.98)	3.59 (3.95)	3.63 (3.99)	3.66 (3.97)
Number of cases (%)	217 (43%)	213 (42%)	209 (41%)	639 (42%)
**Total**	500 (100%)	502 (100%)	502 (100%)	1504 (100%)

The proportion of ‘cases’ (score of 4 or more) in the whole sample was 42%, consistent across groups (Table 2). A chi-square test revealed there were no significant differences in the proportion of ‘cases’ between groups ((χ^2^ (2)n = 1504) = 0.32, p = 0.85).

## Discussion

Our results demonstrate that single errors in GHQ-12 response options do not affect the results. Unexpectedly, even when an error occurred in the first item of the scale, making answers at both the left and right side appear to count as ‘poor wellbeing,’ there was no impact on results. Because the scale was presented to participants on a single screen, they may have observed the tendency across all response sets for the right-hand options to reflect worse wellbeing and deduced how to answer the first item correctly. If true, then our findings may not generalise to equivalent errors in items that do not appear within a scale, or for items in scales where the response sets do have a consistent pattern.

Further, if participants are ‘learning’ how to respond when multiple items are presented simultaneously on a single screen, they could habitually answer negatively or positively rather than thinking about the individual items in their own right. Previous psychometric testing has revealed that the GHQ-12 measures three domains: social dysfunction, anxiety and loss of confidence [10]. As use of the GHQ-12 as a digital survey becomes more common, and given the results of this study, we encourage future research to test the psychometric properties of the measure when presented as multiple items per screen versus single item per screen.

We note that our sample may be limited in that these are individuals who have signed up to market research and have an interest in participating in research generally. These individuals may not be representative of the general population, and therefore, this may limit the generalisability of our findings.

While our data may be reassuring to researchers who, like us, have previously made an error in the options listed for the GHQ-12, they should also be reassuring to those who have made less obvious errors. We reviewed many versions of GHQ-12 available online and elsewhere while preparing this paper, and identified multiple small differences between them. The version used in this study was triple-checked against the original GHQ monograph [11]. Given that completely changing the meaning of a response option seemingly has no effect on estimations of mental health symptoms, it seems likely that smaller alterations such as presenting “less so than usual” as a response option rather than “less able than usual” can be safely ignored.

The results of this study may have implications in settings other than research. Clinicians working in primary care also use and transcribe the GHQ-12 into digital mediums to detect possible mental health problems. If a transcription error caused someone to be wrongly classified as below or above the cut-off for a probable mental disorder, then this could alter an individual’s treatment path. Although the findings of this study do not rule out that one individual will not be impacted by transcription errors, they do offer encouragement that it is unlikely.

## Supporting information

S1 Table Correct and error versions of GHQ-12. (DOCX)
