# Supplementary material for: Do errors in the GHQ-12 response options matter?
Source: PLoS One. 2024 Dec 5;19(12):e0314915. doi: 10.1371/journal.pone.0314915 (PMC11620626; doi:10.1371/journal.pone.0314915)
Supplement: S1 Table — (DOCX) [file pone.0314915.s001.docx]

**S1 Table. Correct and error versions of GHQ-12**

| Correct Item 1 | been able to concentrate on whatever you’re doing? | Better than usual | Same as usual | Less than usual | Much less than usual |
| --- | --- | --- | --- | --- | --- |
| Correct Item 8 | been able to face up to your problems? | More so than usual | Same as usual | Less able than usual | Much less able |
| Error Item 1 | been able to concentrate on whatever you’re doing? | Not at all | Same as usual | Less than usual | Much less than usual |
| Error Item 8 | been able to face up to your problems? | Not at all | Same as usual | Less able than usual | Much less able |
